# Identification of a novel plant polerovirus in the whitefly *Aleuroclava gordoniae*

**DOI:** 10.1128/mra.00900-26

**Published:** 2026-09-08

**Authors:** Hai-Yan Zhu, Jun-Min Li, Hai-Jian Huang

**Affiliations:** 1 Chou Io Insect Museum, Ningbo Yinzhou District Cultural Relics Conservation and Management Center, Ningbo, China; 2 Ningbo Yinzhou District Cultural Center, Ningbo, China; 3 State Key Laboratory for Quality and Safety of Agro-Products, Key Laboratory of Biotechnology in Plant Protection of MARA, Zhejiang Key Laboratory of Green Plant Protection, Institute of Plant Virology, Ningbo University 47862, Ningbo, China; DOE Joint Genome Institute, Berkeley, California, USA

**Keywords:** polerovirus, plant virus, vector insect

## Abstract

We report the genome sequence of *Aleuroclava gordoniae*-associated polerovirus (AgAP), identified from the whitefly *Aleuroclava gordoniae*. The 5,650-nt AgAP genome contains 6 open reading frames. Phylogenetic analysis places AgAP within the genus *Polerovirus*, which comprises plant-infecting viruses. This study provides a genomic resource for further investigation of virus–insect associations.

## ANNOUNCEMENT

The genus *Polerovirus* comprises plant viruses with monopartite, positive-sense RNA genomes of approximately 5–6 kb in length, typically containing six open reading frames (ORFs), designated ORF0 to ORF5 (1). Members of this genus are primarily transmitted by aphids in a circulative, persistent manner (2). An exception is pepper whitefly-borne vein yellows virus, which is transmitted by the whitefly *Bemisia tabaci* (3).

**Fig 1:**
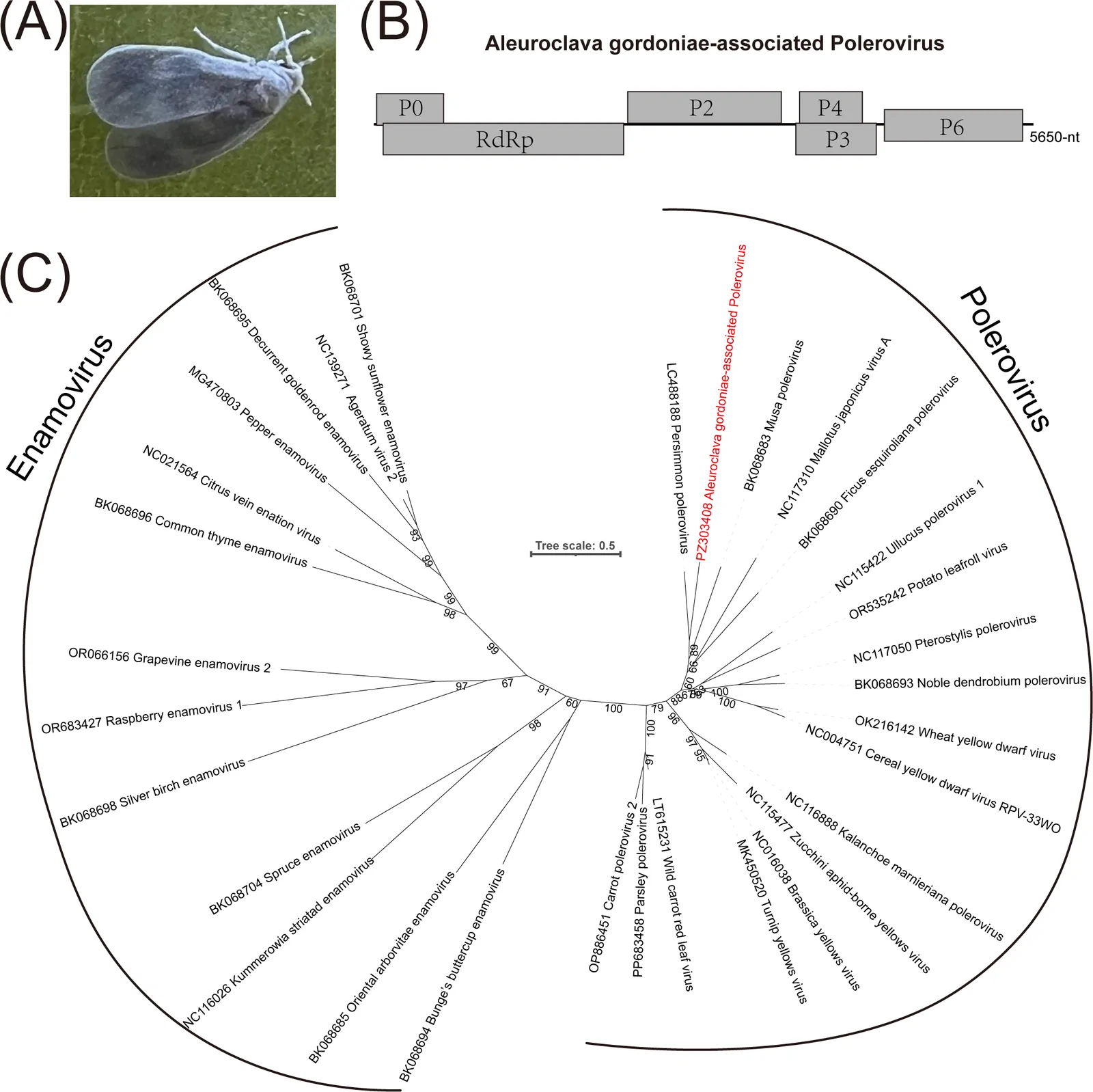
Characterization of *Aleuroclava gordoniae*-associated Polerovirus (AgAP). (**A**) Picture of *Aleuroclava gordoniae* (photographed by Hai-Jian Huang). (**B**) Schematic representation of the AgAP genome. Solid boxes represent putative open reading frames (ORFs). (**C**) Phylogeny of the family Solemoviridae based on RdRP sequences. Amino acid sequences were aligned using MAFFT v7.407 (6). The substitution model was evaluated with ModelTest-NG (7), and a maximum likelihood phylogenetic tree was constructed in RAxML-NG (version 0.9.0) (8), with 1,000 bootstrap replicates. GenBank accession numbers are provided alongside each species. AgAP, identified in this study, is marked in red font.

In this study, we identified a novel polerovirus from the gut of whitefly *Aleuroclava gordoniae* using transcriptomic sequencing of dissected tissues (this virus was not isolated or cultured). Specimens of *A. gordoniae* were collected from *Cinnamomum burmanni* trees in Guilin (25.238° N, 110.175° E), Guangxi, China, in July 2023 (Fig. 1A). The guts and the remaining-body tissues (excluding the gut) were dissected separately in phosphate-buffered saline solution using forceps. No surface sterilization procedure was performed. A total of 10 insects were pooled into 2 samples (gut and remaining-body tissues; no biological replicates). The two samples were homogenized in TRIzol reagent (Takara, Dalian, China) for total RNA extraction. Complementary DNA library construction was performed by Novogene (Tianjin, China) using their standard RNA-seq pipeline (4). Paired-end sequencing (150 bp) was conducted on the Illumina NovaSeq 6000 platform. Raw reads (27,160,830 read pairs for gut sample and 25,133,340 read pairs for remaining-body tissues) were processed using Cutadapt (v1.9) to remove adaptor contamination, low-quality bases, and undetermined bases. Clean read quality was verified using FastQC (v0.10.1) (Q30 >95% for both samples). The filtered reads were not normalized prior to assembly and were subjected to *de novo* assembly using Trinity (v2.6.6) (5), yielding a total of 182,824 contigs (N50 = 1,552; total assembled bases = 152.4 Mb). Default parameters were used except where otherwise noted. All contigs were then searched against the National Center for Biotechnology Information (NCBI) viral RefSeq database (accessed in May 2024) using DIAMOND BLASTx (v2.0.11.149) with an *E*-value threshold of 1 × 10^−20^. A near-complete viral genome of 5,650 nt (GC% = 43.3%; coverage = 655×) was recovered and, based on subsequent phylogenetic analysis, was tentatively named *Aleuroclava gordoniae*-associated polerovirus (AgAP).

Raw reads from both samples were mapped to the AgAP-associated contig using Bowtie2 (v2.5.3) (9) with default parameters. For the gut sample, 24,680 reads (single-end equivalent) mapped to this contig, accounting for approximately 0.09% of the total filtered read pairs, whereas no reads mapped in the remaining-body tissues. The absence of AgAP-derived reads in remaining-body tissues warrants further investigation. Six ORFs were predicted using ORFfinder (www.ncbi.nlm.nih.gov/orffinder) with the standard genetic code and default parameters (Table 1, Fig. 1B). For phylogenetic analysis, representative members of the family Solemoviridae were selected from the NCBI RefSeq database based on taxonomic assignment and availability of full-length RdRP sequences. Phylogenetic analysis of members of the family *Solemoviridae* based on RdRP sequences indicated that AgAP was most closely related to persimmon polerovirus and clusters within the genus *Polerovirus* (Fig. 1C). The near-complete genome of AgAP presented here provides a foundation for future investigations into its biology and ecology.

**TABLE 1:** Predicted open reading frames (ORFs) of AgAP and their best BLAST hits in the NCBI nr database

ORF	Best hit species	Best hit annotation	Best hit accession	Identity	*E*-value
ORF0	*Mallotus japonicus* virus	P0	UWM23161	31.20%	9e−10
ORF1	Persimmon polerovirus	RdRP	BBL52497	38.12%	2e−112
ORF2	Parsley polerovirus	P1-P2 protein	XDE91201	68.68%	0.0
ORF3	Persimmon polerovirus	P3	BBL52499	64.04%	5e−63
ORF4	*Ficus esquiroliana* polerovirus	P4	DBA63698	42.31%	7e−18
ORF5	Persimmon polerovirus	P5	BBL52498	49.08%	2e−46

## Data Availability

The genome sequence of the AgAP genomic RNA has been deposited in GenBank under the accession number PZ303408. The transcriptome raw sequence data are available in the NCBI Sequence Read Archive under accession SRR38125811 and SRR38125812.

## References

[1] LaTourrette K, Holste NM, Garcia-Ruiz H. 2021. Polerovirus genomic variation. Virus Evol 7:veab102. doi:10.1093/ve/veab102 PMC8923251 35299789

[2] Khechmar S, Chesnais Q, Villeroy C, Brault V, Drucker M. 2024. Interplay between a polerovirus and a closterovirus decreases aphid transmission of the polerovirus. Microbiol Spectr 12:e01115-24. doi:10.1128/spectrum.01115-24 PMC11537018 39387567

[3] Ghosh S, Kanakala S, Lebedev G, Kontsedalov S, Silverman D, Alon T, Mor N, Sela N, Luria N, Dombrovsky A, Mawassi M, Haviv S, Czosnek H, Ghanim M. 2019. Transmission of a new polerovirus infecting pepper by the whitefly *Bemisia tabaci*. J Virol 93:e00488-19. doi:10.1128/JVI.00488-19 PMC6639281 31092571

[4] Hu T-B, Wang X-J, Ye Z-X, Lu J-B, Li J-M, Zhang C-X, Huang H-J. 2025. Analysis of salivary proteins in gall-inducing psylla and their potential influence on host plants. BMC Genomics 26:786. doi:10.1186/s12864-025-11958-3 PMC12395826 40877775

[5] Grabherr MG, Haas BJ, Yassour M, Levin JZ, Thompson DA, Amit I, Adiconis X, Fan L, Raychowdhury R, Zeng Q, Chen Z, Mauceli E, Hacohen N, Gnirke A, Rhind N, di Palma F, Birren BW, Nusbaum C, Lindblad-Toh K, Friedman N, Regev A. 2011. Full-length transcriptome assembly from RNA-Seq data without a reference genome. Nat Biotechnol 29:644–652. doi:10.1038/nbt.1883 PMC3571712 21572440

[6] Katoh K, Standley DM. 2013. MAFFT multiple sequence alignment software version 7: improvements in performance and usability. Mol Biol Evol 30:772–780. doi:10.1093/molbev/mst010 PMC3603318 23329690

[7] Darriba D, Posada D, Kozlov AM, Stamatakis A, Morel B, Flouri T. 2020. ModelTest-NG: a new and scalable tool for the selection of DNA and protein evolutionary models. Mol Biol Evol 37:291–294. doi:10.1093/molbev/msz189 PMC6984357 31432070

[8] Kozlov AM, Darriba D, Flouri T, Morel B, Stamatakis A. 2019. RAxML-NG: a fast, scalable and user-friendly tool for maximum likelihood phylogenetic inference. Bioinformatics 35:4453–4455. doi:10.1093/bioinformatics/btz305 PMC6821337 31070718

[9] Langmead B, Salzberg SL. 2012. Fast gapped-read alignment with Bowtie 2. Nat Methods 9:357–359. doi:10.1038/nmeth.1923 PMC3322381 22388286

